# Review of familial hemiplegic migraine, successful outcome in a pregnant patient

**DOI:** 10.1002/ccr3.2513

**Published:** 2019-11-15

**Authors:** Brent C. Monseur, Hannah B. Anastasio, Andrew Haddad, Huda B. Al‐Kouatly

**Affiliations:** ^1^ Department of Obstetrics and Gynecology Sidney Kimmel Medical College of Thomas Jefferson University Philadelphia Pennsylvania; ^2^ Division of Maternal Fetal Medicine Department of Obstetrics and Gynecology Sidney Kimmel Medical College of Thomas Jefferson University Philadelphia Pennsylvania; ^3^ Division of Maternal Fetal Medicine Department of Obstetrics and Gynecology MedStar Washington Hospital Center Washington District of Columbia

**Keywords:** acetazolamide, headache, hormone, reproduction, seizure, stroke

## Abstract

As the field of neurogenetics is expanding rapidly and variant classification criteria evolve, genetic variants in databases are re‐evaluated overtime allowing updated classifications of pathogenicity predication. When caring for patients with genetic disorders, it is important to obtain the original genetic report and also consider an updated reanalysis.

## Graphical abstract

As the field of neurogenetics is expanding rapidly and variant classification criteria evolve, the clinical utility of reanalysis to obtain updated classification of pathogenicity predication is described in a pregnant patient with a diagnosis of familial hemiplegic migraine.

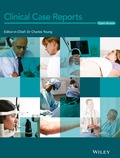

## 1 INTRODUCTION

Familial hemiplegic migraine (FHM) is an autosomal dominant disorder comprised of migraine with aura and associated neurologic deficit, classically motor (ie, hemiparesis). Three genes are described in the literature in relation to FHM: CACNA1A (FHM1), ATP1A2 (FHM2), and SCN1A (FHM3). We report the first successful pregnancy outcome in a woman with FHM. The patient is a 28‐year‐old Caucasian primigravida who transferred care at 29 weeks with a history of FHM and a genetic diagnosis of CACNA1A mutation, reporting 14 years of neurologic symptoms including episodic eye twitching, bilateral weakness, dysarthria, paresthesia, aphasia, and apraxia, lasting from hours to days. She was on acetazolamide which resolved her symptoms. Her care required multidisciplinary approach from maternal‐fetal medicine, reproductive endocrinology, anesthesia, and obstetrics to plan for pregnancy management and delivery. Due to concerns about physical exertion and valsalva with vaginal delivery triggering a symptomatic event, the decision from various teams and the patient was to perform a cesarean section for delivery. Patient had an uncomplicated cesarean delivery following preloading with intravenous fluids prior to spinal anesthesia. A viable female infant was born, and patient had uneventful postpartum course. Upon further review of the genetic report, whole exome sequencing had been performed and a CACNA1A variant was classified as a variant of uncertain significance then. Reanalysis of the CACNA1A reported variant in ClinVar revealed that her mutation is currently classified as benign by several large reference laboratories. We reviewed the pathogenesis of FHM and management options. A multidisciplinary approach resulted in a healthy outcome for the mother and her newborn. In addition, our particular case highlights the importance of not only obtaining the original genetic report but also to consider reanalysis of the genetic results. As the field of neurogenetics expands rapidly, genetic variants in databases are re‐evaluated overtime allowing updated classifications of predicted pathogenicity.

Familial hemiplegic migraine (FHM) is an autosomal dominant disorder comprised of migraine with aura and associated neurologic deficit, classically motor (ie, hemiparesis), with at least one first‐degree relative having identical symptoms.^1^, ^2^ In addition, the aura of FHM may include visual disturbances, sensory loss, dysphasia, and seizures. Symptomatic episodes last hours to days, typically initiating in the first or second decade and decreasing in frequency with age. Three genes are described in the literature in relation to FHM: *CACNA1A* (FHM1), *ATP1A2* (FHM2), and *SCN1A* (FHM3).^1^, ^2^ Our patient presented to our center with diagnosis of FHM due to a mutation in *CACNA1A,* a gene encoding a voltage‐dependent calcium channel, often noted to have a severe phenotype. Severe symptoms of FHM can be managed with acetazolamide or a trial of migraine prophylaxis like beta‐blockers, tricyclic antidepressants, divalproex sodium, calcium channel blockers, and nonsteroidal anti‐inflammatory drugs. Given the rare prevalence of FHM and lack of clinical trials, management therapy is often individualized using a trial‐and‐error strategy.^3^ Due to an increased risk of stroke, vasoconstrictors should be avoided in FHM.^1^, ^2^ We present the first report of a successful pregnancy and delivery in a patient with FHM.

## 2 CASE PRESENTATION

The patient is a 28‐year‐old primigravida who transferred obstetric care to our tertiary care center at 29 weeks of pregnancy due to her history of familial hemiplegic migraine and a genetic diagnosis of a *CACNA1A* mutation.

### 2.1 History

The patient's medical history was otherwise notable for nephrolithiasis, obesity (BMI 38 kg/m^2^), and hyperprolactinemia. Medical records demonstrate 14 years of neurologic symptoms including episodic eye twitching, bilateral weakness, dysarthria, paresthesia, aphasia, and apraxia, lasting from hours to days. Triggers included physical exertion, general anesthesia, and premenstrual hormonal fluctuations. Family history is significant for FHM in the patient's mother. There was no family history of thromboembolic events.

### 2.2 Workup

Migraines can be due to idiopathic intracranial hypertension, central nervous system vasculitis, infarction, as well as neoplastic and non‐neoplastic lesions.^4^, ^5^, ^6^, ^7^, ^8^ This wide differential stresses the importance of both routine and advanced neuroimaging including MRI, which confirmed the absence of pituitary adenoma in consultative reports; however, the original radiology report was not available for our review. In this patient, symptoms were previously misattributed to transient ischemic attacks (TIA) on multiple occasions. The patient's thrombophilia workup was negative. Previous genetic testing confirmed the diagnosis of FHM1 due to CACNA1A mutation.

### 2.3 Treatment

Treatment with acetazolamide resolved her episodic symptoms and was continued daily for prophylaxis. Patient experienced <5 total episodes after initiating therapy. To mitigate premenstrual symptom exacerbation, the patient was on drospirenone/ethinyl estradiol for many years until fertility was desired. Her pregnancy was achieved via in vitro fertilization (IVF). Preimplantation genetic diagnosis was not performed.

### 2.4 Follow‐up during pregnancy

At the time of conception, she had been asymptomatic for 2 years on acetazolamide with her last event occurring after anesthesia for routine esophagogastroduodenoscopy. Prenatal course was notable for the presence of a fetal pelvic kidney and an umbilical vein varix. Sequential screening was low risk for aneuploidy. Fetal echocardiogram was within normal limits. Acetazolamide was self‐discontinued by the patient in the early second trimester due to her concerns about fetal exposure, as well as her lack of symptoms. She remained symptom‐free during pregnancy.

Multidisciplinary input for delivery planning was sought from anesthesiology, neurology, and maternal‐fetal medicine (MFM) to determine the optimal intrapartum management, particularly pertaining to anesthesia, blood pressure, and intravenous fluid management. The patient expressed desire for a primary cesarean section after discussions with her neurologist due to concerns about physical exertion and valsalva with vaginal delivery triggering a symptomatic event.

Preterm premature rupture of membranes occurred at 36 weeks 5 days. An uncomplicated primary cesarean section was performed under spinal anesthesia after preloading with intravenous fluids in order to avoid hypotension and need for vasopressors following regional anesthesia as this could precipitate cerebrovascular accident in this patient. A viable healthy female infant with APGARS of 8 and 9 and a birth weight of 3300 g was delivered.

### 2.5 Follow‐up postpartum

Postpartum course was uneventful, and the mother was discharged home on postoperative day 3 with the neonate, both of whom were in good condition.

At the time of writing, 7 weeks postpartum, the patient was event‐free and had not yet resumed her acetazolamide. Upon further review of the genetic report, whole exome sequencing had been performed and a *CACNA1A* variant was classified as a variant of uncertain significance (VUS) then. Gene sequencing showed a single‐nucleotide substitution from T to G (c.2192) and amino acid substitution from Glu to Ala (p.731) in CACNA1A gene (calcium channelopathy). According to the report, the variant identified is common in the population, highly conserved through evolution, and predicted deleterious by one computer algorithm of protein function and equivocal by another. Due to its population prevalence, this variant is not likely the sole cause of disease in this patient, but a potential risk factor in polygenic disease. With this being the only finding that could explain her symptoms then, she was placed on acetazolamide.

## 3 METHODS

Literature search was performed on 12 July 2018 through the National Center for Biotechnology Information “Gene” database using the search term CACNA1A which identified 226 citations associated with *Homo sapiens *in the PubMed database. We expanded the PubMed search criteria to also include keywords “familial hemiplegic migraine” or “FHM1” and restricted to those with pregnancy‐related terms: (CACNA1A OR FHM1 OR "familial hemiplegic migraine") AND (“reproductive physiological phenomena”[MeSH] OR pregnan* OR labor[tiab] OR delivery[tiab] OR postpartum[tiab] OR antepartum[tiab] OR reproduct*[tiab] OR birth[tiab] OR maternal[tiab]). No limitations were applied for publication date, article type, or language. Out of 27 results, only one relevant publication was encountered.

## 4 DISCUSSION

This is the second pregnancy and first successful pregnancy outcome reported in FHM. Typical migraine triggers such as stress, exertion, food, and odors have been reported to provoke FHM attacks. As observed in our patient, a recent population‐based case‐control study reported increased migraine occurrence in patients using short‐acting benzodiazepines.^9^ Debiais et al reported a 21‐year‐old patient in her second trimester of pregnancy presenting with severe persistent hemiplegic migraine in the setting of a *S218L* mutation associated with severe clinical phenotype. Pregnancy outcome was not discussed in that case report.^10^

Research with cellular and animal models highlights other triggers such as electrolyte imbalance and hormone modulation through increased neuronal excitability and a threshold reduction for spreading depression (SD), a transient succession of waves of electrophysiological hyperactivity and inhibition.^11^ While not clearly elucidated, it is hypothesized that carbonic anhydrase inhibitors, such as acetazolamide, derive their therapeutic effect on channelopathies through effects on potassium levels that affect SD.^12^, ^13^ Of note, acetazolamide has not been associated with birth defects in human pregnancy and is considered compatible with breastfeeding.^14^ Sex hormones, specifically estrogens and progesterone, have also been implicated in pathologic phenotypes associated with SD. This effect is lost in mice after oophorectomy, and androgens are noted to have the opposite effect potentially explaining the increased prevalence of FHM in female mice.^11^ Premenstrual moliminal symptoms including numbness, hand cramps, and a feeling of “disconnectedness” have been reported, as with our patient, and menstrual suppression may mitigate these symptoms. Of note, combined hormonal contraceptives (containing both estrogens and progestins) are categorized as unacceptable health risk in “migraine with aura” of which FHM is a known subtype.^15^ For women of reproductive age planning pregnancy, preconception consultation with maternal‐fetal medicine (MFM) is advised, in addition to genetic counseling. The autosomal dominant mode of inheritance of FHM and 50% risk of an affected child should be reviewed. Options to reduce the risk of transmission include IVF with preimplantation genetic diagnosis, especially if the familial pathologic variant is known, and using oocyte or sperm donor, depending on which parent is affected. Prenatal diagnosis by chorionic villus sampling or amniocentesis should be offered. If an affected patient opts to undergo IVF, special consideration should be made to a regimen that minimizes risk of ovarian hyperstimulation and electrolyte abnormalities.

Pregnancy management of patients with FHM should be multidisciplinary, involving input from MFM, neurology, and anesthesiology. In particular, consultation with anesthesiology prior to the onset of labor is advocated to determine anesthetic plan. Given that vasoconstrictors may precipitate cerebrovascular accidents in patients with FHM, we advocate the use of a slowly dosed epidural, or if spinal anesthesia is planned, judicious preloading with intravenous fluids, in the absence of maternal contraindications, is advocated so as to avoid precipitating hypotension. Preparedness on the labor and delivery for the care of a patient with FHM includes availability of acetazolamide, both oral and intravenous, with route of administration indicated by patient status and as needed for neurologic episodes. There is no literature available to inform mode of delivery in patients with FHM. Although our patient elected for cesarean delivery, vaginal delivery seems permissible, and we recommend shared decision making to determine delivery mode.

What is interesting in our patient is that reanalysis of the CACNA1A reported variant in ClinVar based on current American College of Medical Genetics and Genomics (ACMG) variant classification guidelines revealed that her mutation is currently classified as benign (SIFT, PolyPhen, CADD) by several large reference laboratories. Specifically, it was noted by Exome Aggregation Consortium (ExAC) to have a population frequency in healthy control population not consistent with disease frequency of FHM.^15^, ^16^

## 5 CONCLUSION

Our case highlights the first successful pregnancy outcome in a patient with a clinical diagnosis of FHM. In addition, our particular case highlights the importance of not only obtaining original genetic report but also considering reanalysis of genetic results. As the field of neurogenetics expands rapidly and variant classification criteria evolve, genetic variants in databases are re‐evaluated overtime allowing updated classifications of predicted pathogenicity. Ideally, our patient would have undergone reanalysis of her exome sequencing prior to making her reproductive decisions; however, her sequencing data files were not available due to a closure of the laboratory that performed her original testing. Patient was notified about our reanalysis results and will relay this information to her neurologist.

In conclusion, we described the first case of a successful pregnancy and delivery in a patient with FHM. We reviewed the pathogenesis of FHM and management options. A multidisciplinary approach resulted in a healthy outcome for the mother and her newborn.

## CONFLICT OF INTEREST

On behalf of all authors, the corresponding author states that there is no conflict of interest.

## AUTHORS' CONTRIBUTION

All authors BM, HA, AH and HAK: give final approval of this version to be published and have made substantial contributions in drafting and revising critically for important intellectual content.

## ETHICAL APPROVAL

Not applicable.

## CONSENT FOR PUBLICATION

Consent to publish has been obtained from patient in written form.

## Data Availability

Supporting data can be accessed through medical records at Thomas Jefferson Hospital.
